# Serum indoleamine 2,3-dioxygenase activity is associated with reduced immunogenicity following vaccination with MVA85A

**DOI:** 10.1186/s12879-014-0660-7

**Published:** 2014-12-03

**Authors:** Rachel Tanner, Kristina Kakalacheva, Ellen Miller, Ansar A Pathan, Rod Chalk, Clare R Sander, Tom Scriba, Michelle Tameris, Tony Hawkridge, Hassan Mahomed, Greg Hussey, Willem Hanekom, Anna Checkley, Helen McShane, Helen A Fletcher

**Affiliations:** The Jenner Institute, University of Oxford, Oxford, UK; Department of Neuroinflammation, Institute of Experimental Immunology, University of Zurich, Zurich, Switzerland; Royal Sussex County Hospital, Eastern road, Brighton UK; Centre for Infection, Immunity and Disease Mechanisms, Biosciences, School of Health Sciences and Social Care, Brunel University, Middlesex, UK; Structural Genomics Consortium, University of Oxford, Oxford, UK; Cambridge University Hospitals NHS Foundation Trust, Cambridge, UK; South African Tuberculosis Vaccine Initiative, Institute of Infectious Disease and Molecular Medicine and School of Child and Adolescent Health, University of Cape Town, Cape Town, South Africa; London School of Hygiene and Tropical Medicine, Keppel Street, London, UK; Vaccines for Africa Initiative, Cape Town, South Africa; Division of Community Health, Stellenbosch University, Stellenbosch, South Africa; Metropolitan District Health Services, Western Cape, Government: Health, Cape Town, South Africa

**Keywords:** Indoleamine 2,3-dioxygenase, Tryptophan, Kynurenine, Tuberculosis, Vaccine, MVA85A, BCG, Interferon-γ, LC-MS

## Abstract

**Background:**

There is an urgent need for improved vaccines to protect against tuberculosis. The currently available vaccine Bacille Calmette-Guerin (BCG) has varying immunogenicity and efficacy across different populations for reasons not clearly understood. MVA85A is a modified vaccinia virus expressing antigen 85A from *Mycobacterium tuberculosis* which has been in clinical development since 2002 as a candidate vaccine to boost BCG-induced protection. A recent efficacy trial in South African infants failed to demonstrate enhancement of protection over BCG alone. The immunogenicity was lower than that seen in UK trials.

The enzyme Indoleamine 2,3-dioxygenase (IDO) catalyses the first and rate-limiting step in the breakdown of the essential amino acid tryptophan. T cells are dependent on tryptophan and IDO activity suppresses T-cell proliferation and function.

**Methods:**

Using samples collected during phase I trials with MVA85A across the UK and South Africa we have investigated the relationship between vaccine immunogenicity and IDO using IFN-γ ELISPOT, qPCR and liquid chromatography mass spectrometry.

**Results:**

We demonstrate an IFN-γ dependent increase in IDO mRNA expression in peripheral blood mononuclear cells (PBMC) following MVA85A vaccination in UK subjects. IDO mRNA correlates positively with the IFN-γ ELISPOT response indicating that vaccine specific induction of IDO in PBMC is unlikely to limit the development of vaccine specific immunity. IDO activity in the serum of volunteers from the UK and South Africa was also assessed. There was no change in serum IDO activity following MVA85A vaccination. However, we observed higher baseline IDO activity in South African volunteers when compared to UK volunteers. In both UK and South African serum samples, baseline IDO activity negatively correlated with vaccine-specific IFN-γ responses, suggesting that IDO activity may impair the generation of a CD4+ T cell memory response.

**Conclusions:**

Baseline IDO activity was higher in South African volunteers when compared to UK volunteers, which may represent a potential mechanism for the observed variation in vaccine immunogenicity in South African and UK populations and may have important implications for future vaccination strategies.

**Trial registration:**

Trials are registered at ClinicalTrials.gov; UK cohort NCT00427830, UK LTBI cohort NCT00456183, South African cohort NCT00460590, South African LTBI cohort NCT00480558.

**Electronic supplementary material:**

The online version of this article (doi:10.1186/s12879-014-0660-7) contains supplementary material, which is available to authorized users.

## Background

Tuberculosis (TB) poses an increasing global health threat, with about 10 million new cases and 1.7 million deaths annually (WHO Global Tuberculosis report) [1]. Most of the global TB burden is borne by the developing world, with 86% of cases occurring in South-East Asia, Africa and the Western Pacific. With the emergence of multi-drug resistant strains of *Mycobacterium tuberculosis* (MTB) and the spread of HIV, there is an even greater need for an improved vaccine. *Mycobacterium bovis* Bacille Calmette-Guerin (BCG) is the only vaccine currently available against TB. Although BCG confers reliable protection against disseminated TB during childhood [2],[3], protection against adult pulmonary disease varies considerably with geographical location [4].

A similar population-dependent variability in vaccine immunogenicity has been noted with several other vaccines [5]-[7]. Here we report lower vaccine-specific IFN-γ ELISPOT responses in South African adults when compared with UK adult volunteers following administration of the TB vaccine candidate MVA85A. MVA85A is a recombinant strain of modified vaccinia virus Ankara expressing the immunodominant mycobacterial antigen 85A (Ag85A) from MTB. MVA85A has proven to be both safe and immunogenic [8]-[11]. However, in a recent phase IIb efficacy trial in South African infants immunogenicity was modest and there was no significant protection from clinical disease [11]. In this study we have investigated the relationship between vaccine immunogenicity and the enzyme Indoleamine 2,3-dioxygenase (IDO) in different populations.

IDO catalyses the first and rate-limiting step in the breakdown of the essential amino acid tryptophan (L-Trp) into kynurenine (L-Kyn) and other downstream metabolites [12]. IDO is expressed intracellularly in a constitutive or inducible manner in most non-hepatic cell types, predominantly in the lungs and placenta [13]. Induction is seen in response to various stimuli including IFN-α/β and bacterial lipopolysaccharide, but the most potent inducer is gamma interferon (IFN-γ) [14],[15].

Since it was established that IDO is instrumental in the maintenance of maternal-foetal tolerance by T-cell suppression [16], there has been a growing body of research on its immunoregulatory effects. L-Kyn and other catabolites produced through the action of IDO have been implicated in the suppression of T-cell proliferation and induction of apoptosis [17],[18]. Furthermore, IDO expression has been shown to induce regulatory T cells [19] and inhibit natural killer cells [20].

We hypothesised that levels of IDO may be relevant to MVA85A vaccine immunogenicity as several studies have indicated a role for the enzyme in mycobacterial infection and disease [21],[22]. IDO activity in mice increases following MTB infection in an IFN-γ dependent manner [15]. Furthermore, IDO is important in protection of the granuloma from T-cell attack [23], and is induced following BCG vaccination in mice [24]. It was anticipated that IDO expression and activity might similarly be increased following vaccination with MVA85A.

We demonstrate an IFN-γ dependent increase in IDO mRNA expression in PBMC following MVA85A vaccination. IDO mRNA correlates positively with the IFN-γ ELISPOT response indicating that vaccine specific induction of IDO in PBMC is unlikely to limit the development of antigen specific immunity.

IDO activity in the serum of volunteers from the UK and South Africa was also assessed. There was no change in serum IDO activity following vaccination with MVA85A. However, we saw higher baseline IDO activity in South African volunteers when compared to UK volunteers. This higher IDO activity was unlikely due to latent TB infection as IDO activity was lower in both latently TB infected (LTBI) and uninfected UK adults when compared to South African adults. In both UK and South African adults, baseline serum IDO activity negatively correlated with vaccine-specific IFN-γ responses. The strongest correlation was observed between serum IDO at baseline and the magnitude of the ELISPOT response 6 months following immunisation, indicating that IDO activity may impair the generation of a CD4+ T cell memory response.

## Methods

### Ethics statement

UK participants were recruited under protocols approved by the Oxfordshire Research Ethics Committee (OxREC). South African participants were recruited under a protocol approved by the Medicines Control Council of South Africa, the Human Research Ethics Committees of the University of Cape Town (Cape Town, South Africa) and the Oxfordshire Research Ethics Committee (OxREC). Written informed consent was obtained from all individuals prior to enrolment in the trials. Trials were conducted according to the International Conference on Harmonization - Good Clinical Practice guidelines and are registered at ClinicalTrials.gov.

Peripheral blood mononuclear cells (PBMC) were isolated from buffy coats from healthy PPD positive donors (National Blood Service, Bristol), protocols approved by the Oxfordshire Research Ethics Committee (OxREC).

### Study design and participants

All participants received 5 × 10^7^ plaque forming units (PFU) of MVA85A (Table 1). Participants in all groups were 18 to 50 years of age and seronegative for HIV, hepatitis B and hepatitis C viruses. All participants were previously BCG vaccinated.

**Table 1:** **Demographics of study participants**

Trial registration number	NCT00427830	NCT00456183	NCT00460590	NCT00480558
Country	UK	UK	South Africa	South Africa
No of participants	11	11	10	12
LTBI	-	+	-	+
History of BCG	+	+/−	+/−	+/−
Dose of MVA85A	5 × 10^7^ PFU	5 × 10^7^ PFU	5 × 10^7^ PFU	5 × 10^7^ PFU
Age, median (range)	27 (21–54)	30.5 (20–49)	35.5 (20.7–48.7)	36 (27–49)
Gender, % male	39	83	33.3	75
serum samples available, week (number)	0 (10), 1 (5), 4 (8)	0 (10), 1 (6), 4 (10)	0 (10), 1 (10)	-
PBMC samples available, week (number)	0 (8), 1 (9), 4 (9)	-	-	-

Participants were either negative by *ex vivo* ELISPOT assay for ESAT-6 and CFP-10 or latently infected with MTB (LTBI). Volunteers in the LTBI groups were healthy adults, most of whom had been BCG vaccinated (Heaf test grade II-IV, TST induration ≥10 mm) and LTBI was confirmed by an IFN-γ ELISpot response to the *M.tb* specific antigens ESAT-6 and CFP-10 of >50SFC/million. Participants had normal chest radiographs and no clinical evidence of TB disease.

### Immunological assays

Immune responses were measured by *ex vivo* IFN-γ ELISPOT assay with freshly isolated PBMC as previously described [9]. PBMC (0.3 × 10^6^ per well) were cultured for 18 hours with 8 pools of Ag85A peptides (10 μg/ml each peptide) overlapping by 10 amino acids (Pathan [25]). The Ag85A peptide specific IFN-γ ELISPOT data used in this study has been previously reported by others [8]-[10],[26].

### Cell culture

For the measurement of IDO mRNA expression 5 × 10^5^ freshly thawed PBMCs from trial volunteers were stimulated for 16 hours with 85A peptides or media alone. For blocking experiments PBMC were cultured with or without the addition of 10 μg/ml anti-IFN-γ or 10 μg/ml recombinant human IFN-γ (BD Biosciences). CD14+ cells were depleted from buffy coat PBMC from PPD+ donors using CD14 labeled magnetic beads (Invitrogen).

### IDO mRNA quantification by real time PCR

Half a million freshly thawed PBMCs from trial volunteers were stimulated for 16 hours with Ag85A peptides or media alone. Cells were then pelleted and RNA extracted using the RNeasy Minikit (Qiagen) according to the manufacturer’s instructions. Reverse transcription reaction was performed immediately following extraction with 10 μl of RNA extract using oligo-dT (Eurofins MWG Operon) and the Omniscript Kit (Qiagen). RNase inhibitor, 10 U/μl, (Applied Biosciences) was added to the reactions, which were then incubated for 60 minutes at 37°C. Quantitative PCR (qPCR) was performed with 1 μl of cDNA using QuantiTect mastermix (Qiagen) and 10 pmol/μl of the respective forward and reverse primers designed using PrimerQuest (Integrated DNA Technologies) and ordered from Eurofins MWG Operon. The designed primers were as follows:

HPRT (F:TATGGACAGGACTGAACGTC and R:CTACAATGTGATGGCCTCCC)

IDO (F:CTGCTGGTGGAGGACATG and R:CACAGGAAGTTCCTGTGAG)

qPCR was performed using a Roche LightCycler 480 with the following cycling conditions: initial activation for 15 minutes at 95°C followed by 55 cycles of 15 seconds at 95°C, 20 seconds at 60°C, and 20 seconds at 72°C. PCR products of known concentrations (10^6^ to 10^1^ mRNA copies) were used for the generation of a standard curve for each PCR plate. Analysis was performed with Roche LightCycler 480 software and Ct values were used for conversion to mRNA copy number according to the standard curves. Melting curves were calculated to exclude non-specific binding or primer-dimer formation.

### Liquid chromatography-mass spectrometry

For the measurement of serum IDO activity 5 μl 0.1% formic acid (Sigma Aldrich) containing 30 μg/ml of the internal standard L-Trp-(indole-d_5_) (d_5_-Trp) (GK Gas Products Ltd) was added to 100 μl participant serum, and incubated with 140 μl acetonitrile and 160 μl methanol (both Fisher Scientific) for 20 minutes at −20°C. Samples were then centrifuged at 14000 g for 10 minutes at 4°C and the supernatant removed and vacuum dried. The lyophilised samples were re-suspended in 52.5 μl 0.1% formic acid and centrifuge filtered at 14000 g for 30 minutes at 4°C (10 kDa microcentrifuge filter, Millipore). The filtrate was transferred to 0.3 ml autosampler vials for analysis. A set of four mixed standards was prepared in 0.1% formic acid using twofold serial dilutions of L-Trp (50 μg/ml) and L-Kyn (2.5 μg/ml) (both Sigma-Aldrich), and containing 2 μg/ml of the d_5_-Trp internal standard. External standards were run every 10 samples and four-point calibration curves constructed for each set of samples using the mean values from multiple readings across that trial.

Samples were injected into an Acclaim Pepmap 100 c18 1.0 mm × 150.0 mm column (Thermo) using an Ultimate 3000 nano HPLC pump (Dionex) to generate a post-split flow rate of 3 μl/min. For the first 3.5 minutes, the mobile phase was held at 2% acetonitrile in 2% aqueous formic acid (A). A second solvent mixture (B) containing 2% formic acid in acetonitrile was added, increasing gradually in percentage until 17.5 minutes, when it was held at 80% for 7 minutes at a flow rate of 3 μl/ min for the separation. The column eluent was directed to the electrospray ion source of a HCT Ultra ion trap mass spectrometer (capillary 4 kV, skimmer 40 V, desolvation 300°C, nebulizer 10 psi, drying gas 5 l/min) (Bruker Daltonics). The helium collision gas was at 4 × 10^−6^ Bar, and spectra were acquired in multiple reaction monitoring mode. For the measurement of L-Trp and L-Kyn, the *m/z* 205 and *m/z* 209 precursor ions respectively were selected and fragmented at an amplitude of 1.0 (arbitrary units) to produce monitored product ions at *m/z* 188 for L-Trp and *m/z* 192.2 for L-Kyn. For the measurement of the internal standard d_5_-Trp, the *m/z* 210 precursor ion was isolated and fragmented, producing a product ion at *m/z* 192.1, which was then further fragmented to produce a monitored product ion at *m/z* 150. The complete cycle time was 32.2 minutes. The relative abundance of L-Trp and L-Kyn in the samples was calculated by dividing the MS peak area of the monitored ion by the peak area of the internal standard and converted to a concentration using the external standard curves.

### Statistical analysis

The Mann–Whitney test was used for comparison of median values between groups for IDO mRNA expression. The Wilcoxon Signed-Rank test was used for comparisons of IDO activity between time points, and two-tailed Spearman’s Rank Correlation analyses were used to identify correlations between IDO mRNA, IDO activity and fresh IFN-γ ELISPOT data gathered at the time of the clinical trials. *p* < 0.05 was considered to be statistically significant. Analysis was performed using the software Prism 5 (GraphPad Software Inc, CA) and SPSS 20.0 for Windows (SPSS Inc, Chicago, IL).

## Results

### MVA85A shows reduced immunogenicity in South African adults

**Figure 1:**
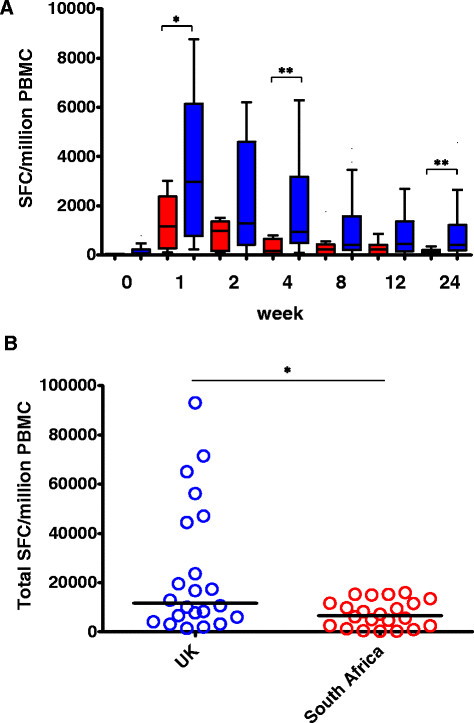
**The immune response following vaccination with MVA85A is lower in South African than UK adults.** Antigen specific IFN-γ ELISPOT responses to Ag85A overlapping peptide pools 0–24 weeks following vaccination with MVA85A are shown. **A)** MVA85A vaccinated adults (red = South African, blue = UK), n = 22 subjects per time point. *indicates a significant difference in response between UK and South Africa at the indicated time point (Mann Whitney Test *P < 0.05, **P < 0.005). **B)** The total immune response following vaccination with MVA85A is lower in South Africa when compared to the UK. Area under the curve (AUC) of the IFN-γ ELISPOT response to Ag85A peptides from 0–24 weeks was calculated for each volunteer. AUC values for each volunteer were then plotted by group (red = South African, blue = UK) n = 22 subjects per group. *indicates significant difference from week 0 (Mann Whitney Test P < 0.05).

We have previously shown that the immunogenicity to MVA85A is not altered by LTBI status within a population, either UK or South African [10],[26]. Data from non-LTBI and LTBI UK populations was therefore combined in to one UK cohort (n = 22) and compared with one combined South African cohort (n = 22). Antigen specific IFN-γ ELISPOT responses to Ag85A overlapping peptide pools were measured 0–24 weeks following vaccination with MVA85A. Immune responses were significantly lower in South African adults at weeks 1, 4 and 24 following vaccination with MVA85A (Mann Whitney Test P < 0.05 or P < 0.005) (Figure 1A). All adults had a history of BCG vaccination and there was an equivalent proportion of LTBI in each cohort (Table 1).

The total immune response following vaccination with MVA85A is lower in South Africa when compared to the UK. Area under the curve (AUC) of the IFN-γ ELISPOT response to Ag85A peptides from 0–24 weeks was calculated for each volunteer and AUC values for each volunteer were plotted by group. The median AUC response was significantly lower in South Africa when compared to the UK (Mann Whitney Test P < 0.05) (Figure 1B).

### IDO mRNA production correlates with IFN-γ response to MVA85A in UK adults, is produced by CD14+ monocytes following vaccination with MVA85A and is dependent on IFN-γ

**Figure 2:**
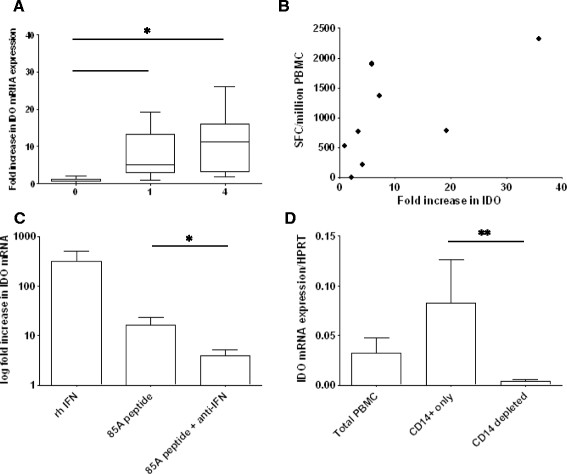
**IDO mRNA following MVA85A vaccination is produced by CD14+ monocytes and dependent on IFN-γ stimulation. A)** PBMC from BCG vaccinated subjects boosted with MVA85A were cultured with 85A peptides or media only and the fold increase in IDO mRNA expression over time was determined for each subject (n = 8). Ag85A-induced IDO mRNA expression was significantly greater at weeks 1 and 4 when compared to week 0 (Wilcoxon *P < .05). **B)** The increase in IDO mRNA expression correlated with the IFN-γ ELISPOT response (data from week 1 following MVA85A) (Spearman’s correlation P < .05, r = 0.79). **C)** PBMC from BCG vaccinated subjects boosted 4 weeks previously with MVA85A were cultured with recombinant human (rh) IFN-γ, 85A peptides or 85A peptides and anti-IFN-γ antibodies and the fold change in expression in stimulated compared to unstimulated PBMC was determined (n = 5). Recombinant human IFN-γ and 85A peptide stimulation induced the expression of IDO mRNA. Co-culturing cells with 85A peptides and anti-IFN-γ antibodies resulted in significant reduction of 85A specific IDO mRNA expression (Wilcoxon *P < .05). **D)** CD14+ magnetic beads were used to deplete monocytes from total PBMC. IDO expression was enriched in the CD14+ fraction and depleted when CD14+ cells were removed (n = 5). Mann Whitney was used for comparison between groups (**P < .005).

We performed quantitative real time PCR analysis to measure the expression of IDO mRNA in PBMC from MVA85A vaccinated adults. PBMC from UK subjects vaccinated with MVA85A were cultured with Ag85A peptides or media only and the fold increase in IDO mRNA expression over time was determined for each subject. Ag85A-induced IDO mRNA expression was significantly greater at weeks 1 and 4 when compared to week 0 (Wilcoxon P < 0.05) (Figure 2A).

The increase in IDO mRNA expression correlated positively with the magnitude of the IFN-γ ELISPOT response following vaccination with MVA85A (Figure 2B). The peak induction of IDO mRNA was observed 4 weeks following immunisation with MVA85A. PBMC isolated 4 weeks following MVA85A vaccination were cultured with recombinant human (rh) IFN-γ, Ag85A peptides or Ag85A peptides and anti-IFN-γ antibodies and the fold change in expression in stimulated compared to unstimulated PBMC was determined. Recombinant human IFN-γ and Ag85A peptide stimulation induced the expression of IDO mRNA. Co-culturing cells with Ag85A peptides and anti-IFN-γ antibodies resulted in a significant reduction of Ag85A specific IDO mRNA expression (Figure 2C). CD14+ magnetic beads were used to deplete monocytes from the PBMC of 5 PPD positive donors. IDO expression was enriched in the CD14+ fraction and depleted when CD14+ cells were removed indicating that CD14+ monocytes are a major source of IDO mRNA production in PBMC cultured with mycobacterial antigens (Figure 2D).

### IDO activity in serum at baseline is higher in South African than UK volunteers

**Figure 3:**
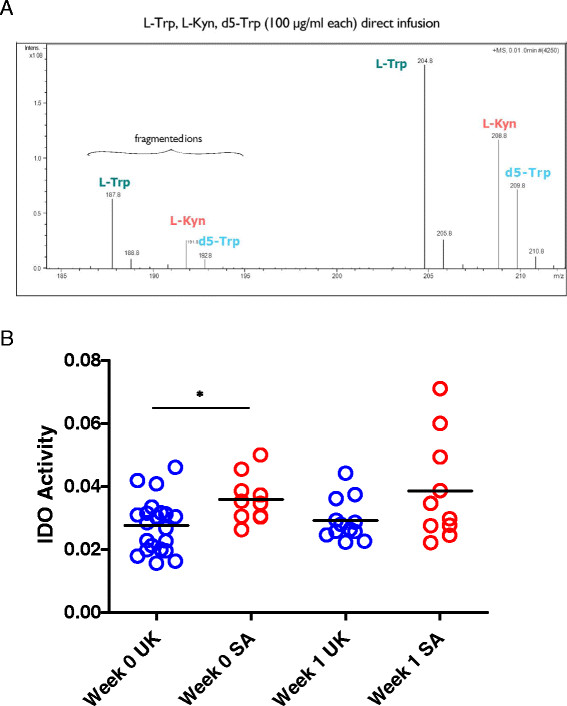
**Serum IDO activity is higher in South African compared to UK adults. A)** Serum IDO activity was measured in serum using LC-MS to quantity L-Trp and L-Kyn in participant serum. The ratio of L-Trp/L-Kyn gives a measure of IDO enzyme activity in host serum. **B)** IDO activity was assessed in serum from UK and South African adults at baseline (pre-vaccination, week 0) and 1 week following immunisation with MVA85A. Mann Whitney was used for comparison between groups (*P < .05).

We used LC-MS to determine L-Trp and L-Kyn levels in the stored sera of UK and South African adults. For the UK volunteers, assays were performed on samples from baseline (pre-vaccination), and weeks 1, 2 and 4 post-vaccination. For the South African group, serum was only available from baseline (pre-vaccination) and week 1 post-vaccination. IDO activity was calculated as the ratio of L-Kyn to L-Trp (Figure 3A). There were no statistically significant differences in levels of L-Trp, L-Kyn or IDO activity pre- and post-vaccination in any individual group, or when all data was combined (Figure 3B). Although there was no vaccine related change in IDO activity, the baseline serum IDO activity in South African volunteers was higher than that of the UK volunteers (*p* = <.05, Mann Whitney) (Figure 3B).

### IDO activity in serum is negatively correlated with the magnitude of the IFN-γ ELISPOT response following vaccination with MVA85A

**Figure 4:**
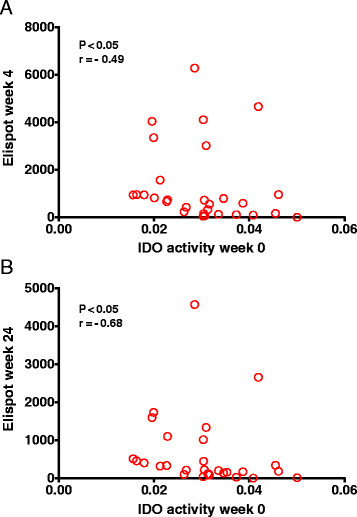
**Serum IDO activity is negatively correlated with the 85A peptide specific IFN-γ ELISPOT response.** IDO activity at baseline for both UK and South African volunteers was correlated with the 85A peptide specific IFN-γ ELISPOT response at **A)** week 4 and **B)** week 24 following vaccination with MVA85A (Spearman’s correlation P < .05).

We correlated IDO activity in serum with *ex vivo* 85A peptide specific IFN-γ ELISPOT responses. In both UK and South African populations IDO activity in the serum was negatively correlated with IFN-γ ELISPOT responses with the strongest correlations seen with IFN-γ ELISPOT responses measured at 4 and 24 weeks (p < .05 and P < .005, Spearman’s) (Figure 4A and B). The relationship between serum IDO activity and the long term ELISPOT response suggests that serum IDO activity may impair the development of a long-term, T cell memory response to Ag85A.

## Discussion

Immune responses to MVA85A were higher in UK adults than South African adults regardless of LTBI status. There was a similar age and gender distribution among the cohorts and all adults were screened to be seronegative for HIV, hepatitis B and hepatitis C. We have previously shown that TGF-β1, regulatory T-cells and TLR-1 expression are correlated with the magnitude of the T-cell response to MVA85A in UK adults [27]-[29]. In this study we have investigated the relationship between vaccine immunogenicity and the enzyme IDO. As MVA85Ax induces potent IFN-γ responses [9], we hypothesised that IDO mRNA would be increased following MVA85A vaccination. In UK adults we observed an increase in IDO mRNA expression at weeks 1 and 4 post-vaccination compared to baseline. IDO mRNA was induced by IFN-γ in CD14+ cells following stimulation of PBMC with Ag85A peptides. To determine if IDO in PBMC could limit the magnitude of the immune response to MVA85A we correlated the increase in IDO mRNA with the Ag85A peptide specific IFN-γ ELISPOT response. IDO mRNA increase correlated positively with the IFN-γ ELISPOT response, suggesting that following vaccination with MVA85A, IDO from CD14+ cells does not limit the secretion of IFN-γ from T-cells.

We then went on to investigate IDO activity in the serum of UK and South African adults using LC-MS. IDO activity in the serum did not change following vaccination with MVA85A, which may be a result of the transient effect of the vaccine; the MVA vector is non-replicating and does not persist in the host and systemic effects are usually undetectable 24–48 hours after immunisation [13],[25].

Although we did not observe a change in serum IDO activity due to vaccination we did see higher baseline IDO activity in the serum of South African adults compared with adults from the UK. This higher IDO activity was unlikely to be due to latent TB infection as IDO activity was lower in both LTBI and uninfected UK adults when compared to South African adults. Active TB patients have increased activity of serum IDO [22], however, we saw no difference in IDO activity by LTBI status, indicating that IFN-γ secretion in response to ESAT-6 and CFP-10 (used here to define LTBI) does not necessarily reflect chronic exposure to MTB or that the exposure is below the threshold required to see a change in systemic IDO activity.

In addition to TB there are other chronic infectious and non-infectious diseases which stimulate the production of IFN-γ and could induce IDO activity. In a recent study of the yellow fever vaccine YF-17D in volunteers from Uganda and Switzerland, Ugandan volunteers demonstrated higher frequencies of exhausted and activated NK cells, differentiated T and B cells, and proinflammatory monocytes at baseline, indicating immune activation [30]. Furthermore, a study of Malawian and UK adolescents showed that Malawian volunteers had a lower percentage of naïve T cells and higher percentage of antigen-experienced T cells and CMV seroprevalence compared with age-matched UK volunteers. The authors conclude that this difference is likely to reflect a greater natural exposure to various infections in the African environment [31]. It may be that a similar effect present in South African volunteers results in constitutively higher levels of IDO activity.

We investigated the relationship between serum IDO activity and the MVA85A vaccine-specific IFN-γ ELISPOT response. Higher IDO activity at the time of MVA85A vaccination is correlated with lower vaccine specific IFN-γ T-cell responses at both 4 and 24 weeks following immunisation, and these are also the time points at which we see the greatest difference in immune response between UK and South African adults (Figure 1). Although the peak effector response to MVA85A is observed 1 week following immunisation, the peak proliferative response is seen at week 24 [32]. IDO has been shown to inhibit both T cell proliferation and the generation of a T cell memory response and it is the longer-term memory response to MVA85A which is most correlated with IDO activity in the serum [17],[33]. In the yellow fever vaccine study described above, Ugandan volunteers showed an impaired vaccine response compared with Swiss volunteers, and this was associated with measures of immune activation at baseline [30].

It is possible that variation in serum IDO activity between populations may also account for variations in the immunogenicity and efficacy of BCG and other T-cell inducing vaccines. Manipulation of this pathway could thus be used to improve vaccine efficacy in endemic countries. There is currently interest in developing IDO inhibitors for the treatment of cancer which may have potential application in vaccination, in particular therapeutic vaccination, as TB patients have high levels of IDO activity. Another strategy is to target the IDO enzyme through boosting of naturally occurring T-cell responses directed against IDO epitopes [34] or silencing of the IDO gene [35].

## Conclusions

Using serum and PBMC collected during a series of phase I trials with MVA85A we have shown that IDO activity in the serum is correlated with the magnitude of the immune response to MVA85A. High baseline IDO activity, possibly resulting from chronic immune activation, could act to reduce IFN-γ ELISPOT responses by limiting T cell proliferation and the development of CD4+ T cell memory. The role of IDO-mediated suppression in vaccine induced immunity warrants further investigation.

## Authors’ original submitted files for images

Below are the links to the authors’ original submitted files for images. Authors’ original file for figure 1 Authors’ original file for figure 2 Authors’ original file for figure 3 Authors’ original file for figure 4

## Abbreviations

MTB: Mycobacterium tuberculosis
TB: Tuberculosis
LTBI: Latently MTB infected
TST: Tuberculin skin test
BCG: Bacille Calmette-Guerin
MVA85A: Modified Vaccinia virus expressing antigen 85A from Mycobacterium tuberculosis
Ag: Antigen
UK: United Kingdom
IDO: Indoleamine 2,3-dioxygenase
L-Trp: Tryptophan
L-Kyn: Kynurenine
IFN-γ: Interferon-gamma
PBMC: Peripheral blood mononuclear cells
mRNA: Messenger ribonucleic acid
ELISPOT: Enzyme-linked immunosorbent spot
PPD: Tuberculin purified protein derivative
ESAT-6: Early secreted antigenic target of 6 kDa
CFP-10: Culture filtrate protein 10
PFU: Plaque forming units
qPCR: Quantitative polymerase chain reaction
LC-MS: Liquid chromatography–mass spectrometry
HPLC: High-performance liquid chromatography
AUC: Area under the curve
