# Supplementary figures and images for: Serum indoleamine 2,3-dioxygenase activity is associated with reduced immunogenicity following vaccination with MVA85A
Source: BMC Infect Dis. 2014 Dec 3;14:660. doi: 10.1186/s12879-014-0660-7 (PMC4265419; doi:10.1186/s12879-014-0660-7)

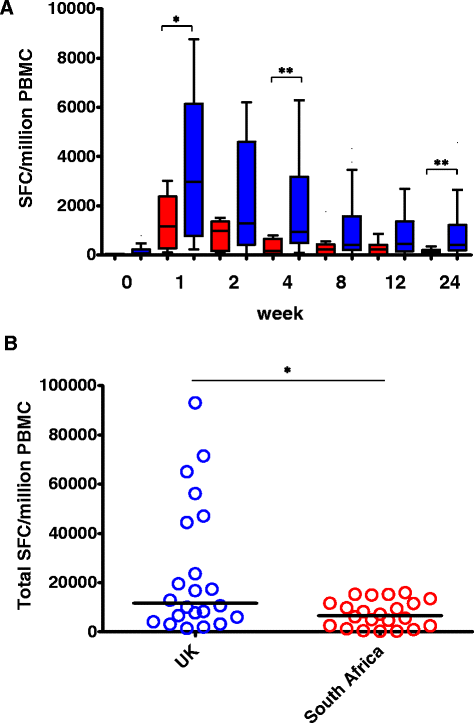

Supplement: Supplementary file 1 — Authors’ original file for figure 1 [file 12879_2014_660_MOESM1_ESM.gif]

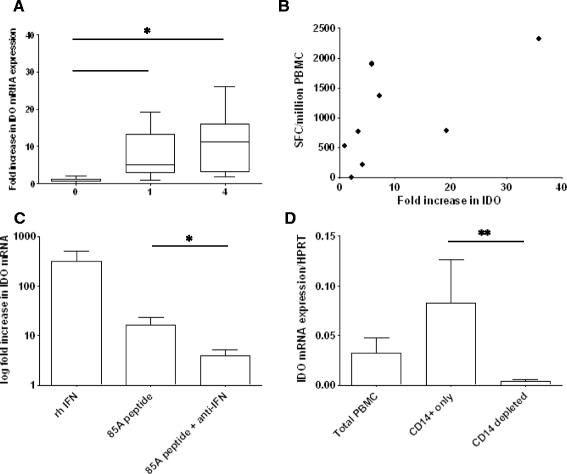

Supplement: Supplementary file 2 — Authors’ original file for figure 2 [file 12879_2014_660_MOESM2_ESM.gif]

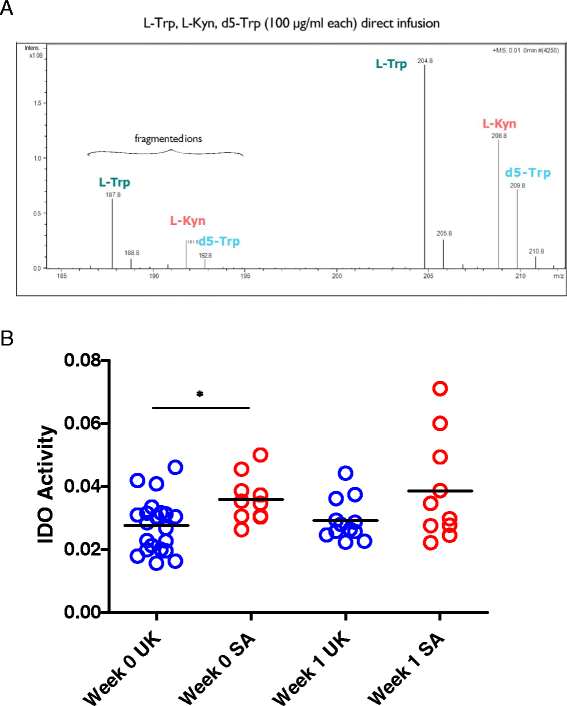

Supplement: Supplementary file 3 — Authors’ original file for figure 3 [file 12879_2014_660_MOESM3_ESM.gif]

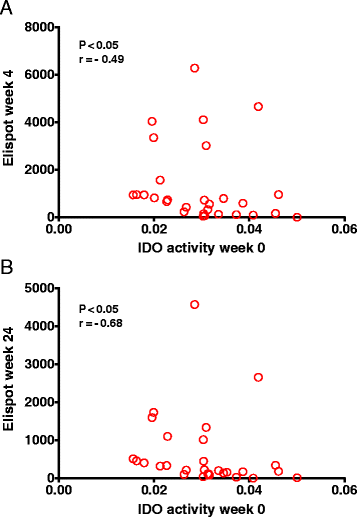

Supplement: Supplementary file 4 — Authors’ original file for figure 4 [file 12879_2014_660_MOESM4_ESM.gif]
